# Quantum crystallographic charge density of urea

**DOI:** 10.1107/S2052252516006242

**Published:** 2016-06-08

**Authors:** Michael E. Wall

**Affiliations:** a Computer, Computational, and Statistical Sciences Division, Los Alamos National Laboratory, Mail Stop B256, Los Alamos, New Mexico 87545, USA

**Keywords:** charge density, quantum theory, spherical atom model

## Abstract

Standard X-ray crystallography methods use free-atom models to calculate mean unit-cell charge densities. Real molecules, however, have shared charge that is not captured accurately using free-atom models. To address this limitation, a charge density model of crystalline urea was calculated using high-level quantum theory and was refined against publicly available ultra-high-resolution experimental Bragg data, including the effects of atomic displacement parameters. The resulting quantum crystallographic model was compared with models obtained using spherical atom or multipole methods. Despite using only the same number of free parameters as the spherical atom model, the agreement of the quantum model with the data is comparable to the multipole model. The static, theoretical crystalline charge density of the quantum model is distinct from the multipole model, indicating the quantum model provides substantially new information. Hydrogen thermal ellipsoids in the quantum model were very similar to those obtained using neutron crystallography, indicating that quantum crystallography can increase the accuracy of the X-ray crystallographic atomic displacement parameters. The results demonstrate the feasibility and benefits of integrating fully periodic quantum charge density calculations into ultra-high-resolution X-ray crystallographic model building and refinement.

## Abstract 2

A charge-density model of urea was calculated using quantum theory and was refined against publicly available ultra-high-resolution X-ray diffraction data. The quantum model differs from a multipole model but agrees comparably with the data; quantum crystallography therefore can provide unique and accurate charge density models.

## 1. Introduction   

Efforts to increase the accuracy of charge density models from X-ray crystallography have mainly focused on fitting the Bragg data using functions that are more expressive than the usual free-atom spherical distributions. Stewart (1969 ▸) proposed using general scattering factors that are the products of atom-centered orbital wavefunctions, and restrictions to better match the number of free parameters to the number of reflections in fitting (Stewart, 1970 ▸). Coppens *et al.* (1971 ▸) separated the free atom charge density into core and valence components, and allowed them to be centered on different positions. Dawson decomposed the charge into symmetric and antisymmetric components centered on each atom (Dawson, 1967*a* ▸), and expanded each atom-centered charge density in spherical harmonics (Dawson, 1967*b* ▸). Hirshfeld developed a least-squares method that models aspherical atomic charge densities using basis functions related to spherical harmonics, but with alternative symmetry properties (Hirshfeld, 1971 ▸). Spherical harmonic-related methods were integrated into multipole refinement computer programs that are used when charge density models are desired (Hansen & Coppens, 1978 ▸; Hirshfeld, 1977*a* ▸; Craven & Weber, 1977 ▸; Stewart & Spackman, 1983 ▸; Jelsch *et al.*, 2005 ▸; Volkov *et al.*, 2006 ▸).

Although less well exploited than multipole methods, the potential for combining quantum theory and X-ray diffraction to obtain accurate charge density models of molecular crystals has been long appreciated (Lipscomb, 1972 ▸). This combination has been termed quantum crystallography (Massa *et al.*, 1995 ▸). The high computational cost of quantum electronic structure calculations has been a major barrier to exploiting the theory for crystallography; however, recent linear scaling methods have made calculations possible for large systems (Bowler & Miyazaki, 2010 ▸, 2012 ▸; Goedecker, 1999 ▸; VandeVondele *et al.*, 2012 ▸), and fast quantum molecular dynamics simulations for systems approaching 10^4^ atoms with 10^5^ time steps are now possible (Mniszewski *et al.*, 2015 ▸). Methods using quantum theory to calculate crystallographic charge density models for all but the largest systems therefore might soon be within reach, not only for small-molecule crystallography (Capelli *et al.*, 2014 ▸) but also for macromolecular crystallography.

Several methods have been proposed for quantum crystallography, including the method of kernel projector matrices (Massa *et al.*, 1995 ▸) and fitting of wavefunctions to diffraction data (Jayatilaka, 1998 ▸). One method that is showing promise in practical applications is Hirshfeld Atom Refinement (HAR) (Bruning & Feil, 1992 ▸; Capelli *et al.*, 2014 ▸; Jayatilaka & Dittrich, 2008 ▸). In HAR, the static charge density of a molecule is calculated using quantum theory and is partitioned into individual atom contributions using Hirshfeld’s stockholder method (Hirshfeld, 1977*b* ▸). The partitioned charge is used to calculate aspherical atomic structure factors that are substituted for the usual structure factors in crystallographic refinement, considering both the atomic positions and displacement parameters (Bruning & Feil, 1992 ▸). Whereas Bruning & Feil (1992 ▸) originally decomposed the charge density into individual atom contributions using a multipole expansion; the more recent implementation of Jayatilaka & Dittrich and coworkers (Capelli *et al.*, 2014 ▸; Jayatilaka & Dittrich, 2008 ▸) directly makes use of a Becke grid for individual atom charge densities. The HAR method has been automated to apply iterative updates of the quantum electronic structure calculation during refinement of atomic positions (Capelli *et al.*, 2014 ▸). So far HAR has been limited to gas-phase electronic structure calculations, with cluster charges placed at symmetry-related positions to approximate the crystal environment.

Whether HAR or other quantum crystallography methods will be adopted widely depends critically on whether they will substantially increase the accuracy of X-ray crystallography models. To date the main focus on the accuracy of HAR has been whether it yields molecular geometry and atomic displacement parameters that are consistent with neutron crystallography. The results here have been promising: applications to X-ray diffraction from crystalline benzene and urea (Jayatilaka & Dittrich, 2008 ▸), l-phenyl­alaninium hydrogen maleate (Woińska *et al.*, 2014 ▸), and a Gly l-Ala dipeptide (Capelli *et al.*, 2014 ▸) found that HAR bond distances agreed very well with neutron crystal structures, overcoming known deficiencies in spherical-atom charge density models (Lipscomb, 1972 ▸). Atomic displacement parameters from HAR similarly agreed reasonably well with the neutron crystal structures.

This study addresses a major factor that so far has been lacking in evaluating quantum crystallographic methods: the accuracy of the charge density model. Here, charge density models for crystalline urea are obtained using spherical atom, atomic multipole or quantum methods. For the quantum method, the HAR method (Jayatilaka & Dittrich, 2008 ▸) is adapted for crystalline phase electronic structure calculations performed using *VASP* (Kresse & Furthmüller, 1996 ▸). Electronic structure calculations using *VASP* previously were performed on hexachlorobenzene for comparisons to the X-ray crystallographic multipole charge density (Aubert *et al.*, 2011 ▸), but without allowing for individual ADPs. The novel aspect of the present method therefore is the combination of a crystalline phase density-functional-theory-based electronic structure calculation with an atomic displacement model from HAR. The results indicate that HAR can yield not only molecular geometries and ADPs that are similar to the neutron crystal structure, but also both 2*F*_o_ − *F*_c_ maps and static charge densities that are distinct from the multipole model, but that nevertheless agree comparably with the experimental data. Quantum crystallography therefore can yield accurate charge densities that are consistent simultaneously with theory and experiment.

## 2. Methods   

### 2.1. Diffraction data and initial crystal structure   

Ultra-high-resolution urea synchrotron diffraction data were obtained from Birkedal *et al.* (2004 ▸) at http://journals.iucr.org/a/issues/2004/05/00/xc5013/xc5013Isup7.hkl. These data were collected at a temperature of 123 K using a wavelength of 0.5996 (1) Å, and were merged into 1045 unique reflections (992 positively valued) extending to 0.347 Å resolution. The data were consistent with a 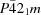 unit cell (space group 113), with *a* = *b* = 5.5780 (6), *c* = 4.6860 (7) Å, α = β = γ = 90°. Other data collection details are published in Birkedal *et al.* (2004 ▸), Table 1 ▸. The multipole refined urea crystal structure was obtained from Birkedal *et al.* (2004 ▸) at http://journals.iucr.org/a/issues/2004/05/00/xc5013/xc5013sup1.cif. The hydrogen parameters of this model were copied from a 123 K neutron crystal structure (Swaminathan *et al.*, 1984 ▸).

### 2.2. Spherical atom and multipole crystallographic models   

The program *SHELXL* (Sheldrick, 2008 ▸, 2015 ▸), Version 2014/1, was used to refine a spherical atom model of urea. Atomic coordinates and anisotropic atomic displacement parameters (ADPs) were refined for all atoms, in addition to an overall scale factor (27 parameters in all). The experimental temperature of 123 K was selected for geometry restraints. *SHELXL* reported agreement factors for the final model are: *R*_1_ = 0.0370, *wR*_2_ = 0.0796, and *SHELX* goodness of fit = 0.639 for all reflections. Mean unit cell charge-density maps *F*_o_, 2*F*_o_ − *F*_c_, and *F*_o_ − *F*_c_ were calculated using the program *shelx2map* provided in the *SHELX* software distribution, using the refined .fcf file as the input, with default weighting, yielding a map of dimensions 56 × 15 × 42 for the asymmetric unit. The maps were expanded to *P*1 using *CCP4* (Stein *et al.*, 1994 ▸) *mapmask* and were interpolated to a 64 × 64 × 64 grid using *CCP4 maprot*.

The program *MoPro*, Version 14.06 (Guillot *et al.*, 2001 ▸; Jelsch *et al.*, 2005 ▸), was used to refine a multipole charge-density model of urea. Refinement was based on the ultra-high-resolution data and structure from Birkedal *et al.* (2004 ▸). 20 cycles of automated density refinement were performed using the REFI DENS method. The total number of free parameters was 37: one scale factor; five valence (VAL); five κ_1_ (K1); five κ_2_ (K2); 21 P_lm_ (PLM) multipole parameters. The atom coordinates and ADPs were kept constant. *MoPro* reported agreement factors for the final model are: *R*_F_ = 0.0242, *wR* ^2^ *F* = 0.0107, *R*_I_ = 0.0212, *wR* ^2^ *I* = 0.0212, and *GooF* = 2.293 for 992 nonzero reflections. Charge density maps were calculated using the *MoPro* supplied program *VMoPro*. The *F*_o_, 2*F*_o_ − *F*_c_, and *F*_o_ − *F*_c_ maps were computed by Fourier reconstruction using the FOUR method using the refined .par file and .FOUR file as inputs, with default resolution limits and the FFT method, yielding maps on a 92 × 92 × 80 grid; these maps were interpolated to a 64 × 64 × 64 grid using *CCP4 maprot* (Stein *et al.*, 1994 ▸). The total static crystalline charge density was computed using the *VMoPro* STAT method, with a 10 Å selection for grid limits, grid-cube dimensions in fractional coordinates, the origin at (0,0,0), a maximum coordinate value of 0.9844 in each dimension, a 10 Å margin around the grid for contributing atoms, and 64 × 64 × 64 grid points. *MoPro* charge densities were scaled to yield a total charge of 64 electrons in the unit cell.

### 2.3. Quantum crystallographic models   

A custom implementation of the original Hirshfeld atom refinement method (Jayatilaka & Dittrich, 2008 ▸) was used to obtain quantum crystallographic models. Quantum charge density calculations were performed using atomic coordinates from each of three different models: the *SHELX* refined spherical atom structure; the neutron crystal structure of Swaminathan *et al.* (1984 ▸); and the multipole model of Birkedal *et al.* (2004 ▸). An expanded unit cell with 16 atoms was generated using the Computational Crystallography Toolbox (*cctbx*) (Grosse-Kunstleve *et al.*, 2002 ▸) by applying 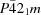 symmetry to the five-atom asymmetric unit. *Ab initio* density functional theory calculations were performed using *VASP*, Version 5.3.3 (Kresse & Furthmüller, 1996 ▸). Instead of pseudopotentials, the PAW method was used, with PAW_PBE parameters (Kresse & Joubert, 1999 ▸). The electronic structure was computed using 4^3^ = 64 Monkhorst-Pack *k* points. Partial occupancies were calculated using Fermi smearing at the experimental temperature of 123 K. As there are fewer than 20 atoms in the expanded urea unit cell, LREAL = .FALSE. was used to evaluate projection operators in reciprocal space, as recommended in the *VASP* documentation. The valence charge density *v*(*x*) was calculated for the expanded P1 unit cell. In addition, using the same *VASP* PAW method as for the molecular calculation, 16 crystalline core charge densities *c_i_*(*x*) and 16 crystalline free-atom (‘promolecule’) charge densities 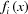 were obtained for each individual atom *i*.

To achieve the desired model accuracy, all *VASP* charge densities were calculated on a 128 × 128 × 128 grid spanning the unit cell. For crystallographic refinement, the densities were decimated to a 64 × 64 × 64 grid. The decimated total static charge density calculated in *VASP* is provided in the supporting information, along with the difference between the *VASP* and *MoPro* multipole static charge density.

X-ray structure factors were calculated using both new and existing tools in *Lunus* software (Wall, 2009 ▸), which was originally designed for analysis and modeling of diffuse X-ray scattering data (Wall *et al.*, 1997*a* ▸,*b* ▸, 2014 ▸). The effect of ADPs was modeled using a Stockholder method (Bruning & Feil, 1992 ▸; Hirshfeld, 1977*b* ▸). The total valence density *v*(*x*) was partitioned into atomic contributions using the equation
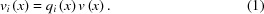
Hirshfeld partitioning (Hirshfeld, 1977*b* ▸) was used with weights 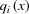 defined using the free-atom charge density 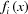


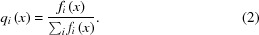
Similar to Bruning & Feil (1992 ▸), ADPs were modeled by treating each partitioned atom charge density 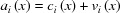 as a rigid distribution, displaced along with the atom. However, in contrast to Bruning & Feil (1992 ▸), instead of using a multipole expansion, the charge density 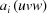 was sampled on a rectilinear grid spanning the unit cell, indexed by *uvw*. This method is similar to that of Jayatilaka & Dittrich (2008 ▸), who used a radial-angular Becke grid for sampling. Here a rectilinear grid is chosen, as it corresponds precisely both to the *VASP* results and to the discrete sampling by the Bragg peaks in the crystallographic experiment. The partitioned atom structure factors were defined as 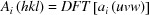, where DFT denotes a discrete Fourier transform. The DFT was computed using a fast Fourier transform (FFT) algorithm (Press *et al.*, 1999 ▸).

In the original Hirshfeld refinement method (Jayatilaka & Dittrich, 2008 ▸), the value 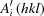 of 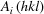 after a coordinate shift 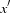 was obtained by multiplying 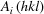 by a phase factor. Although multiplication by a phase factor is appropriate for arbitrary translations of a continuous distribution or an atom-centered grid, it is not appropriate for translations by fractional grid points on a fixed rectilinear grid such as is used here. The correct transformation instead requires a resampling of the shifted distribution 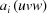 on the original grid (*Appendix* [App appa]). The structure factors are obtained by transforming 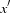 to the grid coordinates 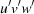, decomposing these coordinates into integer (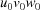) and fractional (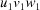) parts such that 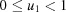, 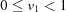, and 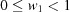, and using the following equation to calculate 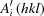


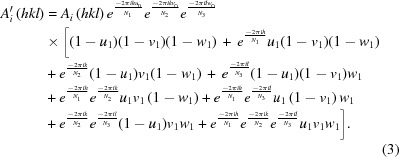
The unit-cell structure factor 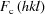 was then calculated as
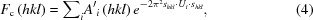
where 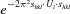 is the Debye–Waller factor for the matrix 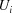 of ADPs for atom *i*, and 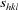 is the scattering vector corresponding to Miller indices *hkl*.

### 2.4. Quantum model refinement   

Quantum refinements were performed starting with the spherical atom (S), multipole (M) (Birkedal *et al.*, 2004 ▸), and neutron crystallography (N) (Swaminathan *et al.*, 1984 ▸) atomic coordinates and ADPs. Model refinement was performed by minimizing the goodness-of-fit (*GooF*) statistic
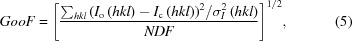
where 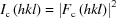, 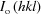 and 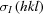 are the values and errors of the observed intensities, and the number of degrees of freedom *NDF* = 965 is the number of data points (= 992 non-negative intensity values in the merged data set), minus the number of free parameters in the fit (= 27, see below). A value of the *GooF* for each set of coordinates and ADPs was obtained by minimizing it with respect to an arbitrary scale factor between the calculated and observed reflection amplitudes. Each matrix 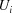 was decomposed into eigenvalues and eigenvectors, and three Euler angles were computed from the eigenvectors, to obtain a set of independent parameters for efficient optimization. Optimization with respect to atom positions and eigenvalues and Euler angles from *U* matrices was performed in python using the *scipy.optimize.minimize* Powell method, using default settings. Due to the use of the Powell method, error bars were not obtained for the fitted parameter values. Eigenvalues were constrained to be positive. Symmetry of the atomic coordinates and ADPs was enforced explicitly using the following equations: 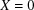 and 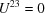 for C, O atoms; 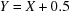 for all atoms; and 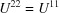 and 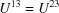 for all atoms. Enforcement of symmetry reduced the number of free parameters from 9 to 4 for the C, O atoms and to 6 for the N, H1, and H2 atoms. There were a total of 27 free parameters in the refinement, including the scale factor between the data and model (the same number as for spherical atom refinement, but without geometry restraints).

The mean unit cell charge densities ρ were calculated using Fourier reconstruction as 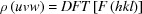. The experimental *F*_o_, 2*F*_o_ − *F*_c_ and *F*_o_ − *F*_c_ maps were calculated by applying the model phases to the observations. Values of 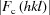 were used in place of missing values of 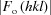. Only complete grids were used for FFT calculations on the quantum models; reflections were not truncated using a resolution cutoff. The *F*_o_, *F*_c_, 2*F*_o_ − *F*_c_, and *F*_o_ − *F*_c_ maps obtained using the multipole model as an input structure are provided in the supporting information.

### 2.5. Agreement factors   

The agreement of all models with the diffraction data was assessed using several standard statistics: *GooF*, *wR* ^2^ *F*, *wR* ^2^ *I*, *RF*, and *RSR*. The *GooF* [equation (5)] was used as the refinement target. The weighted *R*-squared factor for amplitudes, *wR* ^2^ *F*, was calculated as
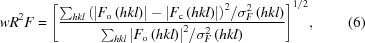
where 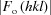 and 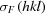 are the experimental reflection amplitudes and errors, and 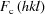 is calculated using equation (4). The weighted *R*-squared factor for intensities, *wR* ^2^ *I*, was calculated as
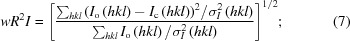
the *R* factor for amplitudes, RF, was calculated as
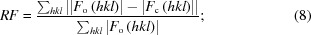
the real-space *R*-factor, RSR, was calculated as
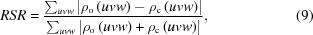
where 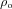 is the experimental *F*_o_ map. Calculated and observed values were scaled to minimize the RMSD prior to using equations (5)–(9) [Disp-formula fd6] [Disp-formula fd7] [Disp-formula fd8] [Disp-formula fd9], and both 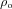 and 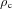 were offset to have zero mean prior to using equation (9). To enable fair comparison, all agreement factors were calculated using *Lunus* software tools. Values reported in primary references were very similar to those computed using *Lunus*.

## 3. Results   

The agreement factors for all quantum crystallographic models are the same (in % units): *wR* ^2^ *F* (target) = 0.9, *wR* ^2^ *I* = 1.8, *RF* = 2.3, *RSR* = 2.1 and *GooF* = 1.7 (Table 1 ▸). These are slightly better than the multipole model, which has values 0.2–0.3% higher for each. The quantum and multipole models agree much better with the data than the spherical atom model (Table 1 ▸).

**Figure 1:**
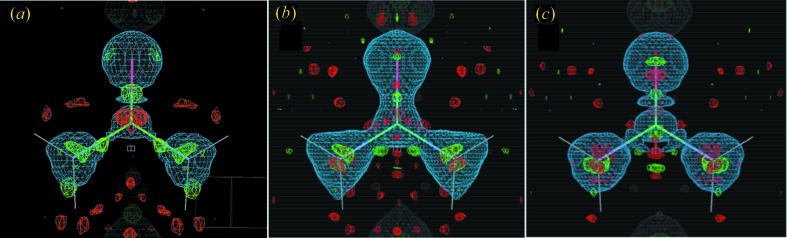
Comparison of 2*F*_o_ − *F*_c_ and *F*_o_ − *F*_c_ maps for (*a*) spherical atom, (*b*) Quantum-M, and (*c*) multipole models. Level charge density surfaces in 2*F*_o_ − *F*_c_ maps are rendered using a blue wireframe at a level of 1-sigma. Level surfaces in *F*_o_ − *F*_c_ maps are shown in green (positive electron density, negative charge) and red (negative electron density, positive charge) wireframes at 3-sigma. The figure was created using *COOT* (Emsley *et al.*, 2010 ▸).

Three-dimensional visualizations of the 2*F*_o_ − *F*_c_ and *F*_o_ − *F*_c_ maps for the spherical atom, quantum-M, and multipole models are shown in Fig. 1 ▸. The spherical atom and multipole 2*F*_o_ − *F*_c_ maps appear to be more similar to each other than they are to the quantum model. This appearance is supported quantitatively using a *RSR* statistic calculated between each pair of 2*F*_o_ − *F*_c_ real-space maps, using an appropriately modified equation (9). A value of 4.9% was obtained between the spherical atom and multipole models. By comparison, the RSR values between the quantum model and either the spherical atom (8.2%) or the multipole model (7.4%) were much greater. These values are all higher than the RSR of any of the models with the data (Table 1 ▸).

**Figure 2:**
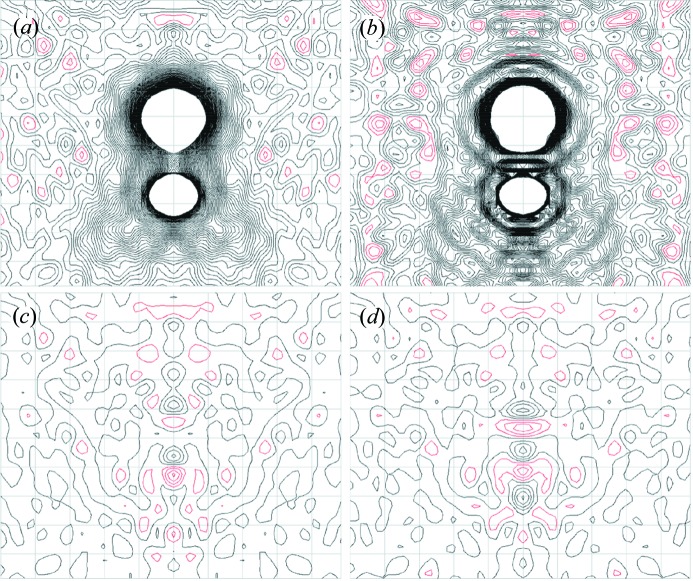
Comparison of two-dimensional contours in the reconstructed mean unit cell charge density for the quantum-M (left), and multipole (right) models in the *y* = 0 section. (*a*)–(*b*) 2*F*_o_ − *F*_c_ maps in 0.05 e Å^−1^ contours, to a maximum of 3 e Å^−1^. (*c*)–(*d*) *F*_o_ − *F*_c_ difference maps in 0.05 e Å^−1^ contours. Negative electron density contours in each panel are colored red. The view is along the same direction as that in Fig. 4 ▸, in the plane of the C=O bond. The orientation is such that *x* increases along the horizontal, and *z* increases along the vertical. The image was created using *mapslicer* in the *CCP4* suite (CCP4, 1994 ▸).

Visualization of contours in a two-dimensional section including the C=O bond reveals that the quantum and multipole 2*F*_o_ − *F*_c_ maps are very different (Figs. 2 ▸ *a* and *b*). (Much of this difference might be an artifact in the multipole map calculation, as mentioned below.) Compared with the multipole model (Fig. 2 ▸ *b*), the quantum-M model is smoother (Fig. 2 ▸ *a*). The multipole model shows ripples surrounding core atoms and peaks away from atoms, including between bonded heavy atoms. The quantum-M model has some peaks away from the atom cores (Fig. 2 ▸ *a*), but these are lower in magnitude compared with the multipole model. The quantum-M and multipole model *F*_o_ − *F*_c_ difference maps are broadly similar (Figs. 2 ▸ *c* and *d*), with the larger deviations from the data in the multipole model along the C=O axis, consistent with the slightly higher values of agreement factors for this model (Table 1 ▸).

**Figure 3:**
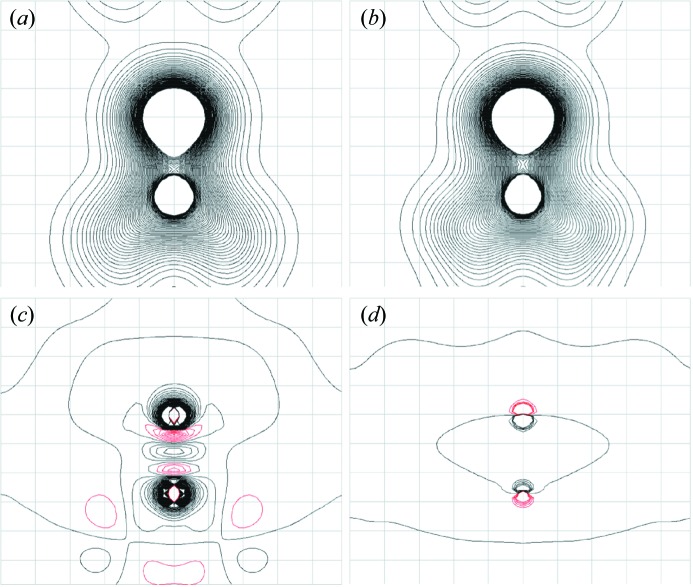
Two-dimensional contours in the static total charge densities derived from the multipole model atom coordinates (Birkedal *et al.*, 2004 ▸) (the section and orientation is the same as in Fig. 2 ▸). (*a*) Theoretical density computed using *VASP*. (*b*) Multipole charge density refinement in *MoPro*. (*c*) Density in (*b*) subtracted from density in (*a*). (*d*) Difference density computed by subtracting the theoretical density using quantum-M refined atom coordinates from the density in (*a*) [the view of the total density using the quantum-M structure is indistinguishable from (*a*)]. Contours in all panels are in 0.05 e Å^−1^ intervals, to a maximum of 3 e Å^−1^. Negative electron density contours in panels (*c*) and (*d*) are colored red. The image was created using *mapslicer* in the *CCP4* suite (CCP4, 1994 ▸).

To investigate further the differences between the 2*F*_o_ − *F*_c_ maps of the quantum-M and multipole models, we compared the static total charge densities calculated using either *VASP* or *MoPro*. Both charge densities correspond to the multipole geometry (Birkedal *et al.*, 2004 ▸). There are visible differences (Figs. 3 ▸ *a* and *b*); however, the differences are much smaller than in the 2*F*_o_ − *F*_c_ maps (Figs. 2*a* and *b*), and they coincide with atoms and bonds. The comparison suggests that the ripples in the 2*F*_o_ − *F*_c_ map from the multipole model are an artifact of the FOUR method implementation in *VMoPro* (*e.g.* a truncation of reflections beyond 0.347 Å resolution).

Subtracting the static charge densities from *VASP* and *MoPro* reveals substantial differences in the charge distribution along the C=O bond (Fig. 3 ▸ *c*). These differences show a similar pattern of peaks and troughs as in the *F*_o_ − *F*_c_ map for the multipole model (Fig. 2 ▸ *d*); by comparison, the *F*_o_ − *F*_c_ map of the quantum-M model shows smaller differences along the C=O bond (Fig. 2 ▸ *c*). Combined, Figs. 2 ▸ and 3 ▸ indicate that the multipole static charge density contains deviations from the data in the C=O bond that are decreased in the quantum-M model.

The *VASP* and *MoPro* calculations were further compared using a Bader analysis of the net atom charges (Tang *et al.*, 2009 ▸; Table 2 ▸). The theoretical *VASP* charges are similar for the spherical atom, multipole and neutron structures. The main difference between these and the multipole model charges is for the C atom, which has a value of 4.06–4.08 electrons from the theoretical density, and 4.57 electrons from the multipole model density. This substantial 0.5 electron difference is compensated by smaller differences in the charges on the other atoms, which are between 0.06 and 0.09 electrons smaller in the multipole model.

The atomic coordinates of the quantum-M, quantum-N, neutron and multipole models are all very similar (Table 3 ▸). The differences between these models and either the spherical atom or quantum-S model are small for the heavy atoms, but are larger for the H atoms. The differences lead to a substantial deviation in the N—H1 bond for the spherical atom and quantum-S structure compared with the neutron structure (Table 4 ▸): the bond length is 1.006 Å in the neutron structure compared with 0.911 Å in the spherical atom and 0.810 Å in the quantum-S structure. The differences also lead to decreases in the C—N—H1 and C—N—H2 bond angles for both the spherical atom and quantum-S structures compared with the neutron structure (Table 5 ▸): the angles in the spherical atom model are about 2° smaller, and the angles in the quantum-S model are about 4° smaller than in the neutron structure. There is a corresponding increase in the H1—N—H2 angle for each compared with the neutron structure: 4° for the spherical atom and 8° for the quantum-S structure. The angle deviations for the quantum-S model are visible in the stick diagram in Fig. 3 ▸(*a*); the effect is smaller but still perceptible for the spherical atom model (not shown).

**Figure 4:**
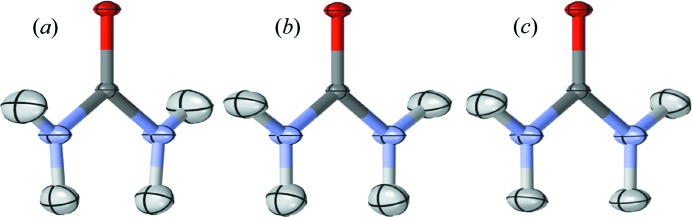
Displacement ellipsoids at 50% probability for (*a*) Quantum-S, (*b*) Quantum-M, and (*c*) neutron diffraction models. In each structure, the O atom is red, the C dark grey, the N blue, and the H1 and H2 light grey, with the H2 at the bottom of the molecule. The Quantum-N model is not shown as it is indistinguishable from the Quantum-M model; similarly, the multipole model is not shown as it is indistinguishable from the neutron. The latter is due to the fact that the hydrogen parameters of the multipole model were copied from the neutron model (Birkedal *et al.*, 2004 ▸). The image was created using *Mercury* (Macrae *et al.*, 2008 ▸).

The similarity of ADPs was assessed using the *S* statistic, which describes the deviation of the three-dimensional positional distribution of the atoms defined by the ADPs (Whitten & Spackman, 2006 ▸). The ADPs for the heavy atoms in the quantum models are very similar to the neutron models (Table 6 ▸; Fig. 4 ▸): the value of *S* for the C atom ranges from 0.04 to 0.06%; the value for the O atom from 0.13 to 0.14%; and the value for the N atom from 0.27 to 0.32%. The similarities are comparable for the multipole model. The ADPs for the H atoms in the quantum models are also similar to the neutron crystal structure, but to a lesser degree than the heavy atoms: the value of *S* for H1 varies from 1.61 to 2.25%; and the value for H2 varies from 2.34 to 3.46%. A high-level quantum theoretical calculation of vibrations of urea to obtain ADPs (Madsen *et al.*, 2013 ▸) yielded a comparable similarity for the heavy atoms (*S* = 0.12, 0.16, and 1.1% for C, O, and N, respectively) and a higher similarity for the H atoms (*S* = 0.13 and 0.05% for H1 and H2, respectively). However, the similarities in Madsen *et al.* (2013 ▸) were computed after applying an overall scale factor with respect to the experimental ADPs; the similarities are considerably lower without applying the scale factor (*S* = 1.36, 0.9, 1.7, 0.5, and 0.65% for C, O, N, H1, and H2, respectively, using the B3LYP/6-31G(d,p) method). The similarity of the spherical atom heavy atom ADPs to the neutron structure is high (*S* = 0.04, 0.13, and 0.21% for C, O, and N), and the similarity for the H atoms is low, as expected for a spherical atom model (*S* = 25.65 and 5.91%). The multipole model hydrogen parameters were copied from the neutron structure and therefore are identical (Birkedal *et al.*, 2004 ▸).

To assess the convergence of the quantum refinement, as was done in previous HAR implementations (Capelli *et al.*, 2014 ▸; Jayatilaka & Dittrich, 2008 ▸), the electronic structure calculation was iteratively applied to each of the quantum models. In the iteration, the refined atomic coordinates were used to re-compute all charge densities using *VASP*, and the model was re-refined against the data using the new densities. The quantum-M and quantum-N models were essentially unchanged in the second iteration: the initial static charge densities were very similar to those for the first iteration, as shown for the quantum-M model in Fig. 3 ▸(*d*); the agreement factors remained the same as in Table 1 ▸; all of the fractional atomic coordinates changed by less than 5 × 10^−3^, with maximal changes of 1 × 10^-3^ for heavy atom coordinates; and the similarity statistic for the ADPs was 0.06% or lower for all atoms between the first and second iterations. In contrast, the quantum-S model showed divergent behavior: the agreement factors were slightly larger (by 0.1–0.2%) for the second iteration; hydrogen fractional coordinates changed by as much as 0.05 (a 0.2 Å shift of the *x*- and *y*-position of the H1 atom); and the similarity statistic for the ADPs was as high as 2.6% (H1 atom), which is comparable to the value computed between the quantum models and the neutron model (Table 6 ▸).

## 4. Discussion   

The agreement of the quantum crystallographic models of urea with ultra-high-resolution data compares favorably to the multipole model. Both the 2*F*_o_ − *F*_c_ map and the total static charge density are substantially different between the quantum and multipole models, however. The differences in 2*F*_o_ − *F*_c_ appear largely to be due to an artifact in the multipole map, as they contain ripples that do not coincide with atom positions or bonds (Figs. 2 ▸ *a* and *b*). The differences in the static charge density, however, appear to be real, with notable differences both in the electronic structure of the C=O bond (Fig. 3 ▸ *c*) and in the 0.5-electron higher negative charge associated with the C atom for the multipole model (Table 2 ▸). The difference in the C atom charge is consistent with the multipole charge density study of Birkedal *et al.* (2004 ▸), which reported a 0.7–0.8 electron larger negative charge for the C atom in the multipole model compared with theoretical charge density calculations.

Whereas Birkedal *et al.* (2004 ▸) concluded the difference between their multipole model of urea and the theoretical charge density was due to inaccuracies in the quantum electronic structure calculation, this study suggests that the difference might instead be due to inaccuracies in the multipole model. The quantum charge densities were obtained using quantum theory and are consistent with the experimental data. The multipole model, although also consistent with the experimental data, is more weakly tied to the underlying theory, and relies on the fitting of many parameters. The possibility of inaccuracies in the multipole model is supported by a controlled study using synthetic data (De Vries *et al.*, 2000 ▸) which found that the charge density of urea could not be determined uniquely using multipole refinement; however, this support is tempered by the fact that the synthetic data did not extend to a resolution as high as the data in Birkedal *et al.* (2004 ▸).

The present results indicate that it would be worthwhile investigating whether HAR might produce more reliable interaction density models than are currently obtained using multipole methods. Compared with multipole refinement, HAR uses fewer parameters and relies on quantum theory for the increased expressiveness needed to model the aspherical component of the charge density. In addition, in HAR, the same quantum electronic structure method used for the crystal phase calculation can be used for the gas phase. Thus, whereas calculating the multipole interaction density involves subtracting two densities that were obtained using substantially different methods, the HAR interaction density can be obtained by subtracting densities that are more comparable.

The thermal ellipsoids in the quantum models are both quantitatively (Table 6 ▸) and qualitatively (Fig. 4 ▸) similar to the neutron crystal structure. This was even the case for the quantum-S model, despite the lack of convergence seen in a second iteration of refinement and deviations in the geometry with respect to the neutron model (Tables 4 ▸ and 5 ▸). This finding is consistent with studies in which the neutron crystallographic temperature factors of urea (Jayatilaka & Dittrich, 2008 ▸) and other systems (Capelli *et al.*, 2014 ▸; Woińska *et al.*, 2014 ▸) were found to be reproduced reasonably well using HAR. In particular, the previous urea study (Jayatilaka & Dittrich, 2008 ▸) used BLYP density functional theory with a cc-pVTZ basis and surrounding charge clusters to mimic periodic boundary conditions, and used the same starting structure as the present quantum-M refinement (Birkedal *et al.*, 2004 ▸). The following ADP values were obtained for H atoms (Jayatilaka & Dittrich, 2008 ▸) (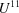, 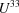, 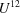, and 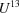 in Å^2^ units): (0.0550, 0.0170, −0.0350, 0) for H1, and (0.0450, 0.0260, −0.0190, −0.0020) for H2. These values are similar to those found here (Table 6 ▸); in addition, values for heavy atoms differed by less than 0.001 Å^2^ compared with those found here. The similarity statistics *S* computed with respect to the ADPs from the quantum-M model are (in % units): 0.01, 0.03, 0.06, 0.26, and 0.08 for the C, N, O, H1, and H2 atoms, respectively. The similarity of the ADPs here with those in Jayatilaka & Dittrich (2008 ▸), in addition to the consistency of the quantum refined ADPs in this study using different starting structures, indicates that HAR can yield estimates of ADPs that are robust to differences in starting structures and DFT methods.

The results for the quantum-M and quantum-N models indicate the potential advantages of quantum crystallography for accurate charge density studies. The lack of convergence and geometry deviations of the quantum-*S* model, however, indicate that challenges remain for the general applicability of these methods. The deviations of the quantum-*S* model can be traced back to deviations in the spherical atom model: although the heavy atoms are consistent with the neutron structure, the deviation in the hydrogen positions is more substantial (Table 3 ▸), leading to corresponding deviations in geometry (Tables 4 ▸ and 5 ▸). These deviations are increased rather than decreased in the quantum-*S* model, which prevents the quantum refinement from converging on successive iterations.

Because it is not currently feasible to obtain neutron crystal structures for all systems of interest, the generalization of the quantum crystallography methods developed here to routine X-ray crystallographic structure determination will require improved modeling of hydrogen positions in the starting structure. The successful application of iterative electronic structure calculations in HAR applications to ammonia and Gly–l-Ala using spherical atoms models like those used here as input structures (Capelli *et al.*, 2014 ▸) indicates that a spherical atom model is adequate for at least some molecular crystals. It would be interesting to determine whether iterative HAR using the implementation of Capelli *et al.* (2014 ▸) converges using the present spherical atom model of urea as an input (as mentioned above, the study of Jayatilaka & Dittrich, 2008 ▸, made use of the same model as the quantum-M refinement here). It is possible that hydrogen positions in spherical atom models would be sufficiently improved using methods that leverage information in structure databases (Bąk *et al.*, 2011 ▸; Bendeif & Jelsch, 2007 ▸; Dadda *et al.*, 2012 ▸; Dittrich *et al.*, 2005 ▸, 2009 ▸), which can place H atoms to within O(10^−2^) Å of the positions in neutron crystal structures.

There are many ways HAR may be extended, targeting, *e.g.*, more accurate models of structure variation than ADPs, and larger systems. For larger systems, it will be especially important to assess the applicability of fast, approximate quantum electronic structure calculations (Mniszewski *et al.*, 2015 ▸) to quantum crystallography. Increasing the speed of calculations would enable the wider adaptation of HAR by adapting existing small-molecule crystallography workflows, and would provide a complementary quantum crystallographic alternative to multipole refinement (Jelsch *et al.*, 2000 ▸) for obtaining high-resolution charge density models of molecular crystals, including macromolecular crystals.

## Supplementary Material

Compressed archive containing charge density maps in CCP4 format.. DOI: 10.1107/S2052252516006242/fc5014sup1.gz

## Figures and Tables

**Table 1:** Values of crystallographic agreement factors for: spherical atom (sphere); quantum starting with the spherical atom (Quant-S), Birkedal *et al.* (2004 ▸) multipole (Quant-M), and neutron (Quant-N) model; and multipole (Birkedal *et al.*, 2004 ▸) model

	*wR* ^2^ *F*	*wR* ^2^ *I*	*RF*	*RSR*	*GooF*
Sphere	0.034	0.068	0.038	0.042	6.5
Quant-S	0.009	0.018	0.023	0.021	1.7
Quant-M	0.009	0.018	0.023	0.021	1.7
Quant-N	0.009	0.018	0.023	0.021	1.7
Multipole	0.011	0.021	0.026	0.023	2.0

**Table 2:** Atom charges based on Bader analysis of total static charge densities *VASP* calculations correspond to the spherical atom (S), multipole (M), and neutron (N) structures. The *MoPro* calculation corresponds to the multipole (M) structure. Units are negative charge in electrons.

	C	O	N	H1	H2
*VASP* (S)	4.08	9.26	8.33	0.51	0.50
*VASP* (M)	4.06	9.26	8.31	0.51	0.53
*VASP* (N)	4.06	9.27	8.31	0.51	0.53
*MoPro* (M)	4.57	9.20	8.22	0.46	0.44

**Table 3:** Coordinates for atoms in the asymmetric unit Model labels in column 2 are as in Table 1 ▸, with the addition of the neutron model (Swaminathan *et al.*, 1984 ▸). Hydrogen model parameters for the multipole model were copied from the neutron model (Birkedal *et al.*, 2004 ▸). Units are fractions of unit-cell dimensions.

		*X*	*Y*	*Z*
C	Sphere	0	0.5	0.3281
	Quant-S	0	0.5	0.3279
	Quant-M	0	0.5	0.3281
	Quant-N	0	0.5	0.3277
	Neutron	0	0.5	0.3280
	Multipole	0	0.5	0.3282
O	Sphere	0	0.5	0.5964
	Quant-S	0	0.5	0.5967
	Quant-M	0	0.5	0.5966
	Quant-N	0	0.5	0.5963
	Neutron	0	0.5	0.5962
	Multipole	0	0.5	0.5963
N	Sphere	0.1450	0.6450	0.1783
	Quant-S	0.1452	0.6452	0.1782
	Quant-M	0.1446	0.6446	0.1796
	Quant-N	0.1447	0.6447	0.1785
	Neutron	0.1447	0.6447	0.1785
	Multipole	0.1447	0.6447	0.1790
H1	Sphere	0.2438	0.7438	0.2793
	Quant-S	0.2316	0.7316	0.2722
	Quant-M	0.2571	0.7571	0.2837
	Quant-N	0.2571	0.7571	0.2838
	Neutron	0.2557	0.7557	0.2841
	Multipole	0.2557	0.7557	0.2841
H2	Sphere	0.1382	0.6382	−0.0363
	Quant-S	0.1324	0.6324	−0.0371
	Quant-M	0.1432	0.6432	−0.0343
	Quant-N	0.1432	0.6432	−0.0344
	Neutron	0.1431	0.6431	−0.0348
	Multipole	0.1431	0.6431	−0.0348

**Table 4:** Bond lengths for alternative models Model labels are as in Table 1 ▸. Units are Å.

	C=O	N—O	N—H1	N—H2
Sphere	1.257	2.268	0.911	1.006
Quant-S	1.259	2.271	0.810	1.013
Quant-M	1.258	2.263	1.011	1.001
Quant-N	1.258	2.266	1.012	0.996
Neutron	1.257	2.266	1.006	1.000
Multipole	1.257	2.265	1.005	1.002

**Table 5:** Bond angles for alternative models Model labels are as in Table 1 ▸. Units are degrees.

	O—C—N	N—C—N	C—N—H1	C—N—H2	H1—N—H2
Sphere	121.54	116.91	117.28	118.42	124.30
Quant-S	121.49	117.04	115.68	115.82	128.50
Quant-M	121.40	117.21	119.87	120.81	119.32
Quant-N	121.49	117.01	119.30	121.02	119.68
Neutron	121.54	116.92	118.99	120.82	120.20
Multipole	121.50	117.01	119.15	120.78	120.07

**Table 6:** Values of ADPs for atoms in the asymmetric unit Labels in column 2 are as in Table 2 ▸. Units are Å^2^. *U* ^11^ = *U* ^22^ and *U* ^13^ = *U* ^23^ by symmetry. The similarity statistic (*S*) with respect to the ADPs of the neutron model is computed following Whitten & Spackman (2006 ▸), in % units. Hydrogen model parameters for the multipole model were copied from the neutron model (Birkedal *et al.*, 2004 ▸).

		*U* ^11^ = *U* ^22^	*U* ^33^	*U* ^12^	*U* ^13^ = *U* ^23^	*S*
C	Sphere	0.0150	0.0070	0.0000	0.0000	0.04
	Quant-S	0.0141	0.0061	0.0001	0.0000	0.05
	Quant-M	0.0141	0.0061	0.0001	0.0000	0.04
	Quant-N	0.0141	0.0060	0.0001	0.0000	0.06
	Neutron	0.0147	0.0065	0.0001	0.0000	0.00
	Multipole	0.0152	0.0068	−0.0004	0.0000	0.03
O	Sphere	0.0199	0.0066	0.0020	0.0000	0.13
	Quant-S	0.0194	0.0059	0.0019	0.0000	0.14
	Quant-M	0.0194	0.0060	0.0020	0.0000	0.14
	Quant-N	0.0194	0.0061	0.0020	0.0000	0.13
	Neutron	0.0197	0.0063	0.0001	0.0000	0.00
	Multipole	0.0196	0.0067	0.0016	0.0000	0.10
N	Sphere	0.0293	0.0096	−0.0155	0.0001	0.21
	Quant-S	0.0285	0.0087	−0.0158	0.0000	0.32
	Quant-M	0.0285	0.0085	−0.0156	0.0001	0.29
	Quant-N	0.0286	0.0087	−0.0156	0.0001	0.27
	Neutron	0.0286	0.0095	−0.0147	0.0002	0.00
	Multipole	0.0293	0.0096	−0.0157	0.0000	0.23
H1	Sphere	0.0295	0.0468	−0.0127	−0.0184	25.65
	Quant-S	0.0550	0.0259	−0.0392	−0.0019	1.61
	Quant-M	0.0495	0.0168	−0.0295	0.0019	2.13
	Quant-N	0.0490	0.0172	−0.0296	0.0022	2.25
	Neutron	0.0440	0.0216	−0.0223	−0.0031	0.00
	Multipole	0.0440	0.0216	−0.0223	−0.0031	0.00
H2	Sphere	0.0415	0.0206	0.0099	−0.0024	5.91
	Quant-S	0.0380	0.0227	−0.0191	0.0015	2.34
	Quant-M	0.0409	0.0270	−0.0187	−0.0013	3.43
	Quant-N	0.0410	0.0272	−0.0186	−0.0012	3.46
	Neutron	0.0430	0.0141	−0.0159	0.0020	0.00
	Multipole	0.0430	0.0141	−0.0159	0.0020	0.00

## Appendix A Transformation of the structure factors upon translation of the charge density

Here the transformation is illustrated using the one-dimensional case; the extension to three dimensions in equation (3) is straightforward. The structure factors are given by the discrete Fourier transform (DFT) of a periodic charge density  sampled at *N* fixed grid points *u*, with . Let the charge density  correspond to the following continuous step-wise distribution

where the Heaviside distribution  for  and  otherwise. Translation of  by a shift  yields a new charge density

Discrete sampling of this shifted charge density on the original fixed grid at integer *u* yields

Decomposing the shift  into an integer component *u*_0_ plus a positive fractional component 0 ≤ *u*_1_ < 1 yields

which is only nonzero for the terms  and . Performing the integrals for these terms yields

Define the structure factors  and .  is obtained from  by applying the DFT shift theorem to equation (14)

which is the one-dimensional version of equation (3). Equation (15) is exact, and demonstrates that  if  includes a nonzero fractional shift .
